# Author Correction: Disrupted sleep-wake regulation in the MCI-Park mouse model of Parkinson’s disease

**DOI:** 10.1038/s41531-024-00746-7

**Published:** 2024-07-15

**Authors:** K. C. Summa, P. Jiang, P. González-Rodríguez, X. Huang, X. Lin, M. H. Vitaterna, Y. Dan, D. J. Surmeier, F. W. Turek

**Affiliations:** 1 Department of Medicine, Feinberg School of Medicine, Northwestern University, Chicago, IL USA; 2 Center for Sleep & Circadian Biology, Northwestern University, Evanston, IL USA; 3 Department of Neurobiology, Weinberg College of Arts and Sciences, Northwestern University, Evanston, IL USA; 4 Department of Neuroscience, Feinberg School of Medicine, Northwestern University, Chicago, IL USA; 5 Department of Molecular & Cell Biology, University of California Berkeley, Berkeley, CA USA; 6 Aligning Science Across Parkinson’s (ASAP) Collaborative Research Network, Chevy Chase, MD 20815 USA; 7 The Ken & Ruth Davee Department of Neurology, Feinberg School of Medicine, Northwestern University, Chicago, IL USA; 8 Department of Psychiatry & Behavioral Sciences, Feinberg School of Medicine, Northwestern University, Chicago, IL USA; 9 Present Address: Neuroscience Discovery, Informatics and Predictive Sciences, Bristol Myers Squibb, Cambridge, MA USA; 10 Present Address: Instituto de Biomedicina de Sevilla, Hospital Universitario Virgen del Rocío/CSIC/Universidad de Sevilla and CIBERNED, Seville, Spain

**Keywords:** Circadian rhythms and sleep, Parkinson's disease

Correction to: *npj Parkinson’s Disease* 10.1038/s41531-024-00670-w, published online 11 March 2024

In this Article the Protocol DOIs were missing and should have been given as follows: “The protocol for sleep-wake recording in the Turek laboratory can be found at 10.17504/protocols.io.yxmvm3z29l3p/v1.” and “The protocol for sleep-wake recording in the Dan laboratory can be found at 10.17504/protocols.io.x54v9pkd4g3e/v1.” The original article and pdf have been corrected.

