# Supplementary material for: Disrupted sleep-wake regulation in the MCI-Park mouse model of Parkinson’s disease
Source: NPJ Parkinsons Dis. 2024 Mar 11;10:54. doi: 10.1038/s41531-024-00670-w (PMC10928107; doi:10.1038/s41531-024-00670-w)
Supplement: Supplementary file 1 — Supplementary Material [file 41531_2024_670_MOESM1_ESM.pdf]

Supplementary Figures

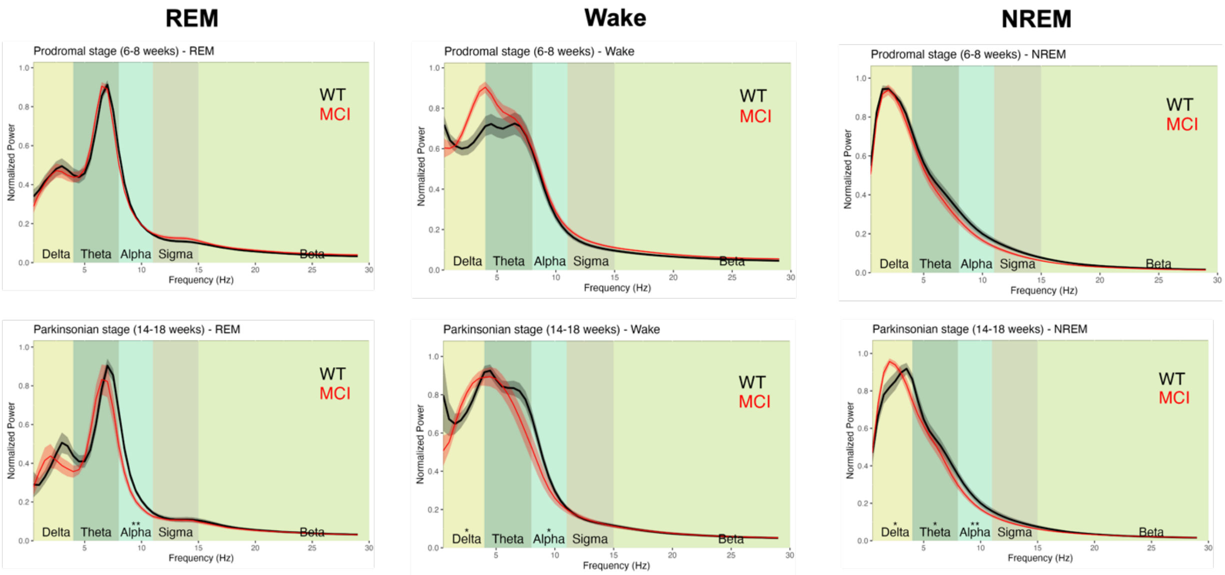

Supplemental Figure 1. EEG power spectral plots for REM sleep (left panel), wake (middle panel), and NREM sleep (right panel) in wildtype (black) and MCI-Park (red) mice at 6-8 weeks of age (top row) and 14-18 weeks of age (bottom row). The EEG power density of the spectrum was first normalized to peak power for each animal, and then the normalized EEG power was averaged across animals within each genotype and age group. PERMANOVA was used to compare different frequency bands within each state, using percentage of total power. *, p<0.05; **, p<0.01. Black and red shading indicate standard error of the mean (s.e.m.) for wildtype and MCI-Park mice, respectively. N=10-23 mice per genotype per age. All data presented in this figure were collected at Northwestern University.

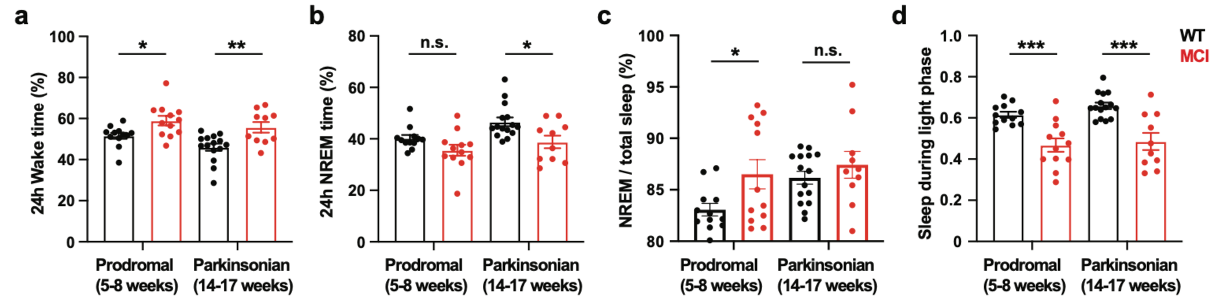

Supplemental Figure 2. Percentage of time spent in wake **(a)** and NREM sleep **(b)** over 24 hours in wildtype (black) and MCI-Park (red) mice at 5-8 weeks of age (left panels) and 14-17 weeks of age (right panels). Proportion of total sleep (NREM plus REM sleep) that is NREM sleep (NREM sleep amount/total sleep amount) **(c)**, and proportion of total sleep during the light phase of the light-dark cycle **(d)** in wildtype (black) and MCI-Park (red) mice at 5-8 weeks of age (left panels) and 14-17 weeks of age (right panels). * $p<0.05$, ** $p<0.01$, *** $p<0.001$; t-test. Each dot represents an individual mouse and error bars depict standard error of the mean (s.e.m.). All data presented in this figure were collected at the University of California, Berkeley.

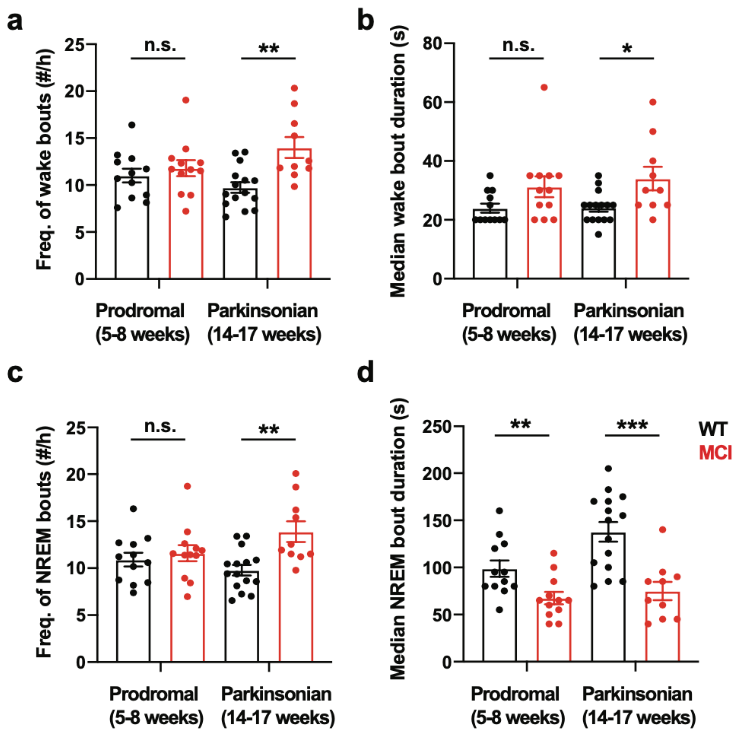

Supplemental Figure 3. Frequency (# bouts/hr) and median duration (s) of bouts of wake **(a, b)** and NREM sleep **(c, d)** in wildtype (black) and MCI-Park (red) mice at 5-8 weeks of age (left panels) and 14-17 weeks of age (right panels). * $p<0.05$, ** $p<0.01$, *** $p<0.001$; t-test. Each dot represents an individual mouse and error bars depict standard error of the mean (s.e.m.). All data presented in this figure were collected at the University of California, Berkeley.

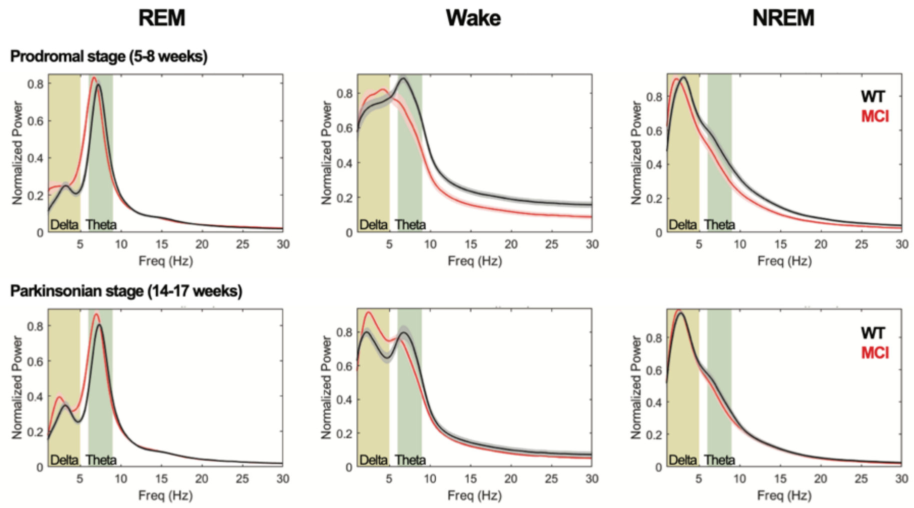

Supplemental Figure 4. EEG power spectral plots for REM sleep (left panel), wake (middle panel), and NREM sleep (right panel) in wildtype (black) and MCI-Park (red) mice at 5-8 weeks of age (top row) and 14-17 weeks of age (bottom row). The EEG power density of the spectrum was first normalized to peak power for each animal, and then the normalized EEG power was averaged across animals. Black and red shading indicate standard error of the mean (s.e.m.) for wildtype and MCI-Park mice, respectively. All data presented in this figure were collected at the University of California, Berkeley.

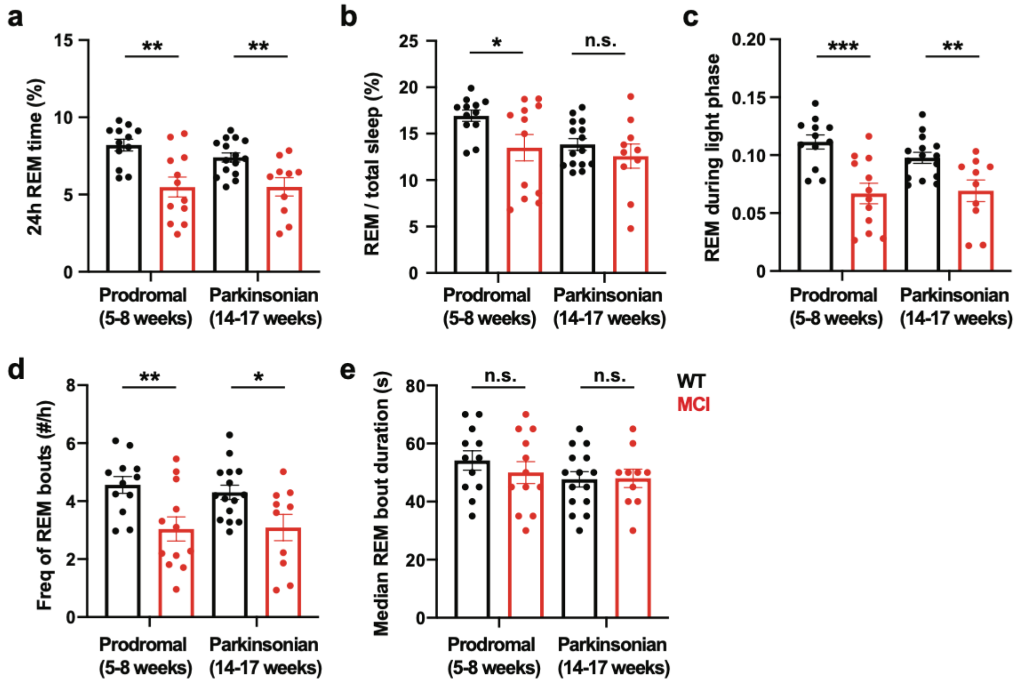

Supplemental Figure 5. Percentage of time spent in REM sleep over 24 hours **(a)**, proportion of total sleep (NREM plus REM sleep) that is REM sleep (REM sleep amount/total sleep amount) **(b)**, proportion of REM sleep during the light phase **(c)**, frequency of REM bouts (# bouts/hr) **(d)**, and median duration of REM bouts (s) **(e)** in wildtype (black) and MCI-Park (red) mice at 5-8 weeks of age (left panels) and 14-17 weeks of age (right panels). * $p<0.05$, ** $p<0.01$, *** $p<0.001$; t-test. Each dot represents an individual mouse and error bars depict standard error of the mean (s.e.m.). All data presented in this figure were collected at the University of California, Berkeley.

Supplementary Methods

Sleep Trait Definitions

%[state]: % of epochs in that state (wake, nrem, rem). %wake + %nrem + %rem + %artifact = 100. %nrem + %rem = %sleep. If an epoch's score is 'unscored' then it will be treated as an artifact.

[state]/sleep: %[state]/%sleep. nrem/sleep + rem/sleep = 1

# [state] bouts: count of the number of bouts of that state

[state] median bout duration: median bout length of that particular state in seconds

brief arousals: the number of single wake epochs in the middle of a sleep bout

state shifts: of the 3 vigilance states (i.e., wake, NREM, or REM), the number of times that the current epoch is not the same as the previous one

[state] [band] total: the average raw value of that band (i.e., delta, theta, alpha, sigma, beta) in that particular state. Excluded epochs are excluded from this calculation.

[state] %[band]: for each epoch in the period of that particular state, a relative power score is determined and expressed as a percentage. That is, if total power = Pt = raw power delta + raw power theta + raw power alpha + raw power sigma + raw power beta, then the relative power of the band = (raw [band]/Pt)*100. The output given is the mean of the relative[band] values. Excluded epochs are excluded from this calculation.
