# Supplementary material for: Disrupted sleep-wake regulation in the MCI-Park mouse model of Parkinson’s disease
Source: NPJ Parkinsons Dis. 2024 Mar 11;10:54. doi: 10.1038/s41531-024-00670-w (PMC10928107; doi:10.1038/s41531-024-00670-w)
Supplement: Supplementary file 2 — Reporting Summary [file 41531_2024_670_MOESM2_ESM.pdf]

nature portfolio

Corresponding author(s): Keith C. Summa

Last updated by author(s): Feb 22, 2024

Reporting Summary

Nature Portfolio wishes to improve the reproducibility of the work that we publish. This form provides structure for consistency and transparency in reporting. For further information on Nature Portfolio policies, see our Editorial Policies and the Editorial Policy Checklist.

Statistics

For all statistical analyses, confirm that the following items are present in the figure legend, table legend, main text, or Methods section.

n/a	Confirmed	
☐	☒	The exact sample size (*n*) for each experimental group/condition, given as a discrete number and unit of measurement
☐	☒	A statement on whether measurements were taken from distinct samples or whether the same sample was measured repeatedly
☐	☒	The statistical test(s) used AND whether they are one- or two-sided<br>*Only common tests should be described solely by name; describe more complex techniques in the Methods section.*
☐	☒	A description of all covariates tested
☐	☒	A description of any assumptions or corrections, such as tests of normality and adjustment for multiple comparisons
☐	☒	A full description of the statistical parameters including central tendency (e.g. means) or other basic estimates (e.g. regression coefficient) AND variation (e.g. standard deviation) or associated estimates of uncertainty (e.g. confidence intervals)
☐	☒	For null hypothesis testing, the test statistic (e.g. *F*, *t*, *r*) with confidence intervals, effect sizes, degrees of freedom and *P* value noted<br>*Give P values as exact values whenever suitable.*
☒	☐	For Bayesian analysis, information on the choice of priors and Markov chain Monte Carlo settings
☒	☐	For hierarchical and complex designs, identification of the appropriate level for tests and full reporting of outcomes
☒	☐	Estimates of effect sizes (e.g. Cohen's *d*, Pearson's *r*), indicating how they were calculated

Our web collection on statistics for biologists contains articles on many of the points above.

Software and code

Policy information about availability of computer code

Data collection	No software was used
Data analysis	Software programs previously described in the published literature were used to assist with the classification of sleep-wake states based on automated analysis of EEG/EMG signals. References to these programs were provided in the text of the Methods section. In addition, the reference identification code and link to a custom-written package on GitHub was provided in the text of the Methods section.

For manuscripts utilizing custom algorithms or software that are central to the research but not yet described in published literature, software must be made available to editors and reviewers. We strongly encourage code deposition in a community repository (e.g. GitHub). See the Nature Portfolio guidelines for submitting code & software for further information.

Data

Policy information about availability of data

All manuscripts must include a data availability statement. This statement should provide the following information, where applicable:

- Accession codes, unique identifiers, or web links for publicly available datasets
- A description of any restrictions on data availability
- For clinical datasets or third party data, please ensure that the statement adheres to our policy

The datasets on sleep-wake traits in MCI-Park and littermate wildtype mice generated in this study are publically available (Northwestern University: DOI:10.5281/zenodo.10079840; University of California, Berkeley: DOI:10.5281/zenodo.10046587). In addition, specific aspects of the raw and processed data will be made

available by the corresponding author (KCS, Northwestern University data) or co-first author (XH, University of California, Berkeley data) in response to reasonable requests from qualified researchers (i.e., researchers affiliated with a university, academic medical center, research institute, or similar institution), pending approval of all senior authors (MHV, YD, DJS, FWT).

Research involving human participants, their data, or biological material

Policy information about studies with human participants or human data. See also policy information about sex, gender (identity/presentation), and sexual orientation and race, ethnicity and racism.

Reporting on sex and gender	This research did not involve human participants, their data, or their biological material.
Reporting on race, ethnicity, or other socially relevant groupings	This research did not involve human participants, their data, or their biological material.
Population characteristics	This research did not involve human participants, their data, or their biological material.
Recruitment	This research did not involve human participants, their data, or their biological material.
Ethics oversight	This research did not involve human participants, their data, or their biological material.

Note that full information on the approval of the study protocol must also be provided in the manuscript.

Field-specific reporting

Please select the one below that is the best fit for your research. If you are not sure, read the appropriate sections before making your selection.

☒ Life sciences ☐ Behavioural & social sciences ☐ Ecological, evolutionary & environmental sciences

For a reference copy of the document with all sections, see nature.com/documents/nr-reporting-summary-flat.pdf

Life sciences study design

All studies must disclose on these points even when the disclosure is negative.

Sample size	Sample size was determined primarily based on the extensive experience recording sleep-wake behavior in laboratory rodents in our laboratories. Considerations included using a minimum number of animals in each experimental condition to detect significant and meaningful differences in sleep-wake traits between different experimental groups while using an adequate number of animals to ensure the validity and reproducibility of the findings.
Data exclusions	The sleep-wake data from one older mouse were excluded from analysis due to a significant number of epochs (>5%) that were unable to be accurately scored into a particular sleep-wake state due to substantial artifact of the EEG and EMG tracings that precluded classification. EEG spectral data from one mouse was excluded because of artifact from the EEG lead used to generate EEG spectral profiles from all other animals. Both of these data exclusions and the rationale for doing so were disclosed in the Methods section of the manuscript.
Replication	The verify the reproducibility of the findings described in this manuscript, separate cohorts of animals were evaluated with consistent results observed. In addition, the studies were independently replicated at a separate institution with consistent results observed. These aspects of replication are discussed in the manuscript.
Randomization	Animals were allocated into experimental groups based on their genotype (wildtype vs mutant) and their age (younger vs older). All animals were subjected to the same sleep-wake recording procedures and analysis so there was no randomization (i.e., there were no treatment and control groups).
Blinding	The sleep-wake data was analyzed in a blinded manner, such that the manually assisted sleep-wake scoring was performed by investigators that were not aware of the experimental group of the animal from which the data file they were scoring was generated.

Reporting for specific materials, systems and methods

We require information from authors about some types of materials, experimental systems and methods used in many studies. Here, indicate whether each material, system or method listed is relevant to your study. If you are not sure if a list item applies to your research, read the appropriate section before selecting a response.

Materials & experimental systems

n/a	Involved in the study
☒	☐ Antibodies
☒	☐ Eukaryotic cell lines
☒	☐ Palaeontology and archaeology
☐	☒ Animals and other organisms
☒	☐ Clinical data
☒	☐ Dual use research of concern
☒	☐ Plants

Methods

n/a	Involved in the study
☒	☐ ChIP-seq
☒	☐ Flow cytometry
☒	☐ MRI-based neuroimaging

Animals and other research organisms

Policy information about studies involving animals; ARRIVE guidelines recommended for reporting animal research, and Sex and Gender in Research

Laboratory animals	The study used laboratory mice obtained from breeding colonies at Northwestern University and University of California, Berkeley. Mutant (MCI-Park) and wildtype littermate control mice were used in the studies as described in the Methods.
Wild animals	Wild animals were not used in the research described in this manuscript.
Reporting on sex	Male and female mice were used in roughly equal numbers in the experiments described in this manuscript. Statistical analyses did not identify any significant differences due to sex for any of the sleep-wake traits examined. Male and female animals were therefore grouped together in subsequent analyses and display items (figures, tables). This was described in the Methods section of the manuscript text.
Field-collected samples	The study did not use any field-collected samples.
Ethics oversight	All studies described in this manuscript were reviewed and approved in advance by the Institutional Animal Care and Use Committees of Northwestern University and the University of California, Berkeley. This is described in the Methods section of the manuscript.

Note that full information on the approval of the study protocol must also be provided in the manuscript.

Plants

Seed stocks	Not applicable
Novel plant genotypes	Not applicable
Authentication	Not applicable
